# The Therapeutic Effects of a Bentonite‐Based Facial Mask With *Alcea sulphurea* Extract on Acne Severity and Patient Experience: Add‐On Randomized Controlled Clinical Trial

**DOI:** 10.1111/jocd.70586

**Published:** 2025-12-05

**Authors:** Sara Salimi, Moloud Fakhri, Majid Saeedi, Seyyed Mobin Rahimnia, Armaghan Kazeminejad, Mahmood Moosazadeh, Emran Habibi, Assie Jokar

**Affiliations:** ^1^ Student Research Committee Mazandaran University of Medical Sciences Sari Iran; ^2^ Traditional and Complementary Medicine Research Center, Addiction Institute Mazandaran University of Medical Sciences Sari Iran; ^3^ Department of Pharmaceutics, Faculty of Pharmacy Mazandaran University of Medical Sciences Sari Iran; ^4^ Pharmaceutical Sciences Research Center, Institute of Herbal Medicines and Metabolic Disorders Mazandaran University of Medical Sciences Sari Iran; ^5^ Department of Dermatology, Faculty of Medicine Mazandaran University of Medical Sciences Sari Iran; ^6^ Gastrointestinal Cancer Research Center, Non‐Communicable Disease Institute Mazandaran University of Medical Sciences Sari Iran; ^7^ Medicinal Plants Research Centre, Institute of Herbal Medicines and Metabolic Disorders Mazandaran University of Medical Sciences Sari Iran; ^8^ Centre for Natural Products Discovery, School of Pharmacy and Biomolecular Sciences Liverpool John Moores University Liverpool UK; ^9^ Department of Persian Medicine. Faculty Of Persian Medicine Mazandaran University of Medical Sciences Sari Iran

**Keywords:** acne vulgaris, add‐on randomized controlled clinical trial, *Alcea sulphurea* extract, bentonite, traditional Persian medicine

## Abstract

**Background:**

Acne vulgaris adversely impacts quality of life, requiring safer alternatives to conventional treatments. This study explores a facial mask combining Bentonite's detoxifying and *Alcea sulphurea*'s anti‐inflammatory properties, rooted in Traditional Persian Medicine (TPM). It aims to evaluate its efficacy in reducing acne severity and enhancing patient satisfaction, integrating traditional and modern therapeutic approaches.

**Methods:**

This add‐on randomized, controlled clinical trial included 60 patients with moderate acne, recruited through purposive sampling in Sari from October 2022 to November 2023. Participants were randomly allocated into intervention (Bentonite‐Alcea mask + Azithromycin 250 mg) and placebo (placebo + Azithromycin 250 mg). Outcomes, including lesion count, acne severity, and satisfaction, were assessed at baseline, 4 weeks, and 8 weeks. Data analysis was conducted using SPSS version 16.

**Results:**

Sixty people completed the study (30 persons in both group). The mean and standard deviation of the age in control group was 24 ± 8.03 years and in placebo group was 19.03 ± 7.03 and 46 subjects (76.7%) were female. TLC and ASI in the fourth and eighth weeks in control group were significantly less than in placebo group (*p* < 0.001). The percentage of relatively satisfied and very satisfied participants in the intervention group was significantly higher than the control group (*p* < 0.001). Bentonite‐based facial mask with *Alcea sulphurea* extract had no serious side effects. The intervention group exhibited significant reductions in TLC (total lesion count) (55.45% ± 21.74%) and ASI (Acne Severity Index) (66.33% ± 27.15%) from baseline to 8 weeks (*p* < 0.001), outperforming the placebo group. Patient satisfaction was notably higher in the intervention group, with 43.3% being very satisfied compared to 3.3% in the placebo group (*p* < 0.001).

**Conclusion:**

A facial mask based on Bentonite and *A. sulphurea* extract effectively reduces acne severity and enhances patient satisfaction. These findings suggest its potential as a natural alternative or adjunct to conventional acne treatments.

**Trial Registration:**

Iranian registry: IRCT20220403054395N1

**Abbreviations**
ASI: Acne Severity Index
GAGS: Global Acne Grading System
TLC: total count lesion
TPM: Traditional Persian Medicine
VAS: Visual Analog Scale

## 1 Introduction

Acne vulgaris is one of the most common dermatological conditions worldwide, affecting individuals across various age groups, particularly adolescents and young adults [1]. Characterized by the presence of comedones, papules, pustules, nodules, and, in severe cases, scarring, acne significantly impacts patients' quality of life, self‐esteem, and mental health [2]. Despite its multifactorial etiology involving hormonal imbalances, microbial colonization, sebum production, and inflammatory processes, the exact mechanisms contributing to acne pathogenesis remain complex and not entirely understood [3].

In the modern medical framework, acne treatment primarily includes topical therapies such as retinoids, benzoyl peroxide, and antibiotics, as well as systemic medications like oral antibiotics, hormonal therapies, and isotretinoin for severe cases [4]. Although these interventions are effective, their side effects, such as skin irritation, antibiotic resistance, and teratogenic risks, limit their universal acceptability. This has prompted researchers to explore complementary and alternative therapies, especially those rooted in traditional medicine, which are often perceived as safer and more holistic [5].

Traditional Persian Medicine (TPM), an ancient system of medicine with roots in Persian philosophy and natural sciences, offers valuable insights into the etiology and management of acne [6]. According to TPM, acne, referred to as “Busoor” or “Gharab,” results from an imbalance in the body's humoral system, particularly an excess of heat and moisture in the blood and skin. This imbalance can be triggered by factors such as poor diet, hormonal fluctuations, and environmental conditions. According to TPM, treatments aim to restore balance by using natural remedies, dietary modifications, and lifestyle changes to reduce inflammation and improve skin health. Among the myriad remedies, clay‐based formulations like Bentonite and plant extracts such as *Alcea sulphurea* have been traditionally utilized for their purported detoxifying, anti‐inflammatory, and emollient properties [7, 8, 9].

Bentonite, also known as Fuller's Earth or Gel‐e‐Sarshour, is a naturally occurring clay with a long‐standing history in dermatological applications due to its ability to absorb excess oil, unclog pores, and gently exfoliate the skin. Its mineral‐rich composition provides a soothing effect, making it particularly suitable for acne‐prone skin [10, 11]. On the other hand, *Alcea* extract is valued for its anti‐inflammatory and mucilaginous properties, which help hydrate the skin, reduce irritation, and promote healing [12, 13]. The combination of these two natural ingredients may offer a potential approach to addressing both the cosmetic and therapeutic aspects of acne management, although further research is needed to confirm their synergistic effects.

Despite the widespread use of such traditional remedies, there is a paucity of rigorous clinical studies evaluating their efficacy and safety in acne treatment. Most existing literature focuses on the individual effects of natural compounds, with limited exploration of their combined applications in formulations tailored to acne patients [6, 14, 15, 16].

By integrating traditional knowledge with modern clinical methodologies, this research seeks to establish a scientific basis for the use of TPM‐based therapies in acne management. Furthermore, the study addresses the growing demand for evidence‐based, natural skincare solutions that are both effective and well‐tolerated. The findings of this study may provide a foundation for the development of innovative, accessible, and sustainable acne treatments that resonate with the preferences of patients seeking alternatives to conventional therapies.

In summary, this study explores a novel TPM‐inspired formulation combining Bentonite and hydroalcoholic extract of *A. sulphurea* , aiming to offer a natural and effective solution for acne vulgaris. By examining its impact on clinical outcomes and patient satisfaction, the research highlights the potential of traditional remedies in complementing modern dermatological practices and contributing to a more holistic approach to acne management.

## 2 Materials and Methods

### 2.1 Overview

This add‐on randomized controlled clinical trial aimed to evaluate the efficacy of a facial mask containing Bentonite and hydroalcoholic extract of *A. sulphurea* on total lesion count (TLC), the acne severity index (ASI), and patient satisfaction in individuals with moderate acne. From October 2022 to November 2023, 60 patients were recruited from eligible referrals in the city of Sari and randomly assigned to either the treatment or placebo group. The study population consisted of acne patients who visited the dermatology clinic at Bu‐Ali Hospital, Tooba Clinic, and Tooba Health Center. This study was approved by the ethics committee of Mazandaran.

University of medical sciences [session no. IR.MAZUMS.REC1400.683]. The protocol adhered to the ethical guidelines of the Declaration of Helsinki. All the participants were fully aware and informed about the goals and details of the study through an information sheet and received informed consent from them. This study was registered in the Iranian registry of clinical trials (https://irct.ir).

### 2.2 Material Preparation

The primary ingredients for the intervention were Bentonite and *A. sulphurea* extract. Bentonite was procured from Germany Merc company and flowers of *A. sulphurea* were procured from a medicinal plants (Exir Company) located in Sari, Mazandaran, Iran. Bentonite was procured from a certified source and processed to remove impurities, ensuring cosmetic‐grade quality. The flowers of *A. sulphurea* were authenticated by a pharmacognosist. After drying at controlled temperatures, the roots were ground and macerated in ethanol to extract bioactive compounds. The extract was filtered, concentrated under reduced pressure, and stored at 4°C.

### 2.3 Standardization of Extracts and Microbial Testing

The *A. sulphurea* extract was standardized based on its total phenolic and flavonoid content, measured using spectrophotometric methods (Folin–Ciocalteu for phenols and aluminum chloride colorimetric assay for flavonoids) [17]. Bentonite was standardized by assessing its mineral composition and absorbency properties using X‐ray fluorescence (XRF) and water absorption tests, ensuring consistency across batches [10]. In addition to the chemical standardization, microbial testing was performed to assess the safety and quality of both the *A. sulphurea* extract and Bentonite. The microbiological analysis followed standard protocols to ensure the absence of harmful microbial contamination, including bacteria and fungi, confirming that the extracts and the clay were safe for use in the formulation.

### 2.4 Drug Preparation

The formulation of the study intervention (facial mask) was carefully designed to ensure both efficacy and patient acceptance. Based on traditional Persian medicine sources, *A. sulphurea* and Bentonite are commonly combined in equal proportions for topical applications. In this study, a paste formulation was selected to serve as a facial mask, providing ease of use and optimal adherence to the skin. To prepare the final drug formulation, a 10% w/w hydroalcoholic extract of flowers of *A. sulphurea* was mixed with Bentonite in a 1:1 ratio. Additional excipients, such as corn starch, glycerin, and water, were incorporated to enhance the textural properties and spreadability, as well as to achieve the total weight required for the mask formulation. To ensure stability and extend the shelf life of the product, preservatives such as sodium benzoate (1% w/w) were added. For the placebo formulation, all nonactive components of the main formulation were included, but *A. sulphurea* and Bentonite were replaced with neutral excipients to mimic the texture and appearance of the active formulation. For the preparation of the traditional medicine product (facial mask), after procuring high‐quality raw materials, the final formulation is prepared by the pharmaceutics experts in the research team.

Azithromycin 250 mg tablet was procured from Loqman Daru Company. Due to the standardization of the commercially available Azithromycin tablets, this formulation is used for patient administration.

### 2.5 Patients

Participants were recruited from patients diagnosed with moderate acne based on the Global Acne Grading System (GAGS). Inclusion criteria included individuals aged 12 years and up with 20–50 total lesions (comedones, papules, pustules) and no nodules or cysts. Exclusion criteria encompassed pregnancy, hormonal disorders like polycystic ovary syndrome, use of systemic acne treatments within 3 months, and known hypersensitivity to the mask components.

### 2.6 Sample Size

According to the Central Limit Theorem, a sample size of 30 or more is generally sufficient for the sampling distribution of the mean to approximate normality. Therefore, we conducted our study with 30 participants in each group (placebo and intervention).

Additionally, we verified normality to ensure appropriate statistical methods are applied [18]. Among the qualified clients, 60 patients were randomly included in the study and divided into two treatment and placebo groups.

### 2.7 Randomization, Allocation Concealment, and Blinding

After selecting eligible participants based on inclusion criteria, randomization was performed using Random Allocation Software, which will create 10 blocks of six participants each. The participants were then assigned to either the intervention or placebo group. To ensure allocation concealment, treatment sequences were written on 60 cards, sealed in opaque envelopes, and labeled with a random four‐digit code. The research assistant had access to the envelopes, but the investigator and data analyst remained blinded to group allocation until the study's completion [19].

Due to the nature of Bentonite, it was not possible to formulate it into a gel with *A. sulphurea* extract. Therefore, the final formulation of the drug was in the form of a mask, while the placebo was in the form of a gel. However, the drug was in an opaque container, and the method of its use and the explanations given to the patient were the same in both groups.

### 2.8 Study Intervention

Azithromycin was a standard treatment for moderate acne. One group received a standard treatment of Azithromycin (as commercially available tablets) combined with a placebo, while the other group received Azithromycin (tablet) along with the traditional medicine product. The medication was applied once daily, before bedtime, as a thin layer on the affected area of the face for 8 weeks. After 10 min, the face was washed with water. Participants were evaluated at Weeks 4 and 8, with the total number of papules, pustules, and comedones assessed. All assessments were conducted by a single investigator.

### 2.9 Study Outcomes

The primary outcomes of the study were as follows:

TLC: The number of acne lesions, including comedones, papules, and pustules, was assessed using the TLC method. This index provides a comprehensive count of all acne lesions present on the skin, reflecting the overall severity of acne.

ASI: The ASI was calculated to assess the severity of acne, using the formula:

$$\mathrm{ASI}=\text{papules}+\left(\text{pustules}\times 2\right)+\left(\text{comedones}÷4\right)$$

The reduction in ASI was categorized as follows: a decrease of less than 30% was considered a poor response, a decrease between 30% and 60% was classified as a moderate response, and a reduction of more than 60% was considered an excellent response.

The secondary outcome included:

*Patient satisfaction*: This was measured using a Visual Analog Scale (VAS), where patients rated their satisfaction with the treatment. The VAS is a reliable tool for evaluating subjective experiences, such as treatment satisfaction, on a scale from 0 (no satisfaction) to 10 (extremely satisfied).

### 2.10 Statistical Analysis

Data analysis was conducted using SPSS version 27 Description of variables with number, percentage, mean, standard deviation, median, IQR, minimum, maximum and range of changes are provided. The normality of quantitative variables was evaluated by the Kolmogorov–Smirnov test. Comparison of grouped variables between the two groups (intervention and placebo) was done using chi‐squared tests (*χ* ^2^) and Fisher's Exact Test. To compare continuous variables between the two groups, independent *t*‐tests and Mann–Whitney tests were used. Moreover, to compare the TLC and the ASI over time (base line times Weeks 4, 8) between the two groups and within each group, repeated measures analysis of variance (ANOVA) was employed. Also, due to the differences in age and education variables between the two groups at baseline, repeated measures ANOVA with covariate was used to control for their confounding effect. For all statistical tests, a *p*‐value less than 0.05 was considered statistically significant.

## 3 Results

### 3.1 Overall Descriptive Statistics

**FIGURE 1:**
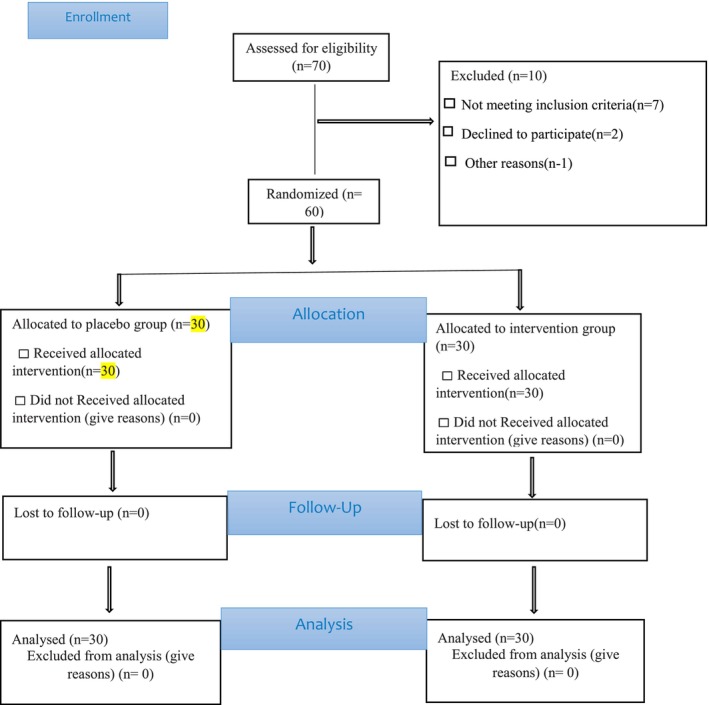
CONSORT flow diagram.

A total of 70 patients with moderate acne vulgaris were evaluated (Figure 1). Finally, 60 patients participated and completed the study (30 patients in the intervention group and 30 patients in the placebo group). During the 8‐week study duration, none of the patients dropped out of the study. Baseline characteristics were assessed to ensure the validity of subsequent comparisons between the intervention and placebo groups. Table 1 summarizes the demographic and clinical features of the participants, including mean age, gender distribution, education level, employment status, body mass index, and the presence of underlying conditions. While most baseline characteristics were comparable between the two groups, no significant differences were observed in gender distribution or baseline severity of acne (*p* > 0.05). However, significant differences were noted in age (*p* = 0.002) and education level (*p* = 0.035).

**TABLE 1:** Baseline demographic characteristics of study groups.

Characteristics	Intervention (*n* = 30)	Placebo (*n* = 30)	*p*
**Age (mean ± SD)**	24 ± 8.03	19.03 ± 7.03	0.002^a^
**Gender [*n* (%)]**			
Male	8 (26.7%)	6 (20.0%)	0.542
Female	22 (73.3%)	24 (80.0%)	
**Level of education [*n* (%)]**			
High school or lower	14 (46.7%)	22 (73.3%)	0.035^a^
Above high school	16 (53.3%)	8 (26.7%)	
**Employment [*n* (%)]**			
Employed	8 (26.7%)	3 (10.0%)	0.095
Unemployed	22 (73.3%)	27 (90.0%)	
**Body Mass Index (BMI)**	23.51 ± 2.59	23.09 ± 2.67	0.542
**Underlying conditions [*n* (%)]**			
Yes	1 (3.3%)	0 (0.0%)	1.000
No	29 (96.7%)	30 (100.0%)	

*Note:* The Kolmogorov–Smirnov test was used to evaluate the normality. According to this test, whenever the significance level of a change is greater than 0.05, that change can be from a normal distribution.

^a^ *p*‐values calculated using independent *t*‐tests for continuous variables and chi‐squared tests for categorical variables.

The flowchart illustrates the patient enrollment process, including screening, randomization, allocation to intervention and placebo groups, and follow‐up throughout the study period. It highlights the number of participants at each stage and ensures transparency in the study design and methodology (Figure 1).

### 3.2 Primary Outcomes

The intervention group demonstrated a steeper decline in TLC and ASI compared to the placebo group, highlighting the efficacy of the natural facial mask in addressing key aspects of acne pathogenesis. Table 2 compares TLC and ASI between the two groups at three time points: baseline, the 4th week, and the 8th week. In the intervention group, both TLC and ASI significantly decreased over time, with mean percent changes of 55.45% and 66.33%, respectively, from baseline to the 8th week (*p* < 0.001). In contrast, the placebo group demonstrated less marked improvements, with percent changes of 21.74% for TLC and 27.15% for ASI during the same period. The reductions in both outcomes were significantly greater in the intervention group. This demonstrates the superior therapeutic potential of the facial mask formulation.

**TABLE 2:** Comparison of TLC and ASI between the intervention and placebo groups over time.

Variable	Group	Beginning (mean ± SD)	4th Week (mean ± SD)	8th Week (mean ± SD)	*p*	*p*	*p*	Effect size *d* (8th vs. beginning)	Percent change^d^ (mean ± SD)
TLC	Intervention	35.63 ± 14.61	21.17 ± 9.95	15.87 ± 10.12	< 0.001^a^	0.018^b^	0.063^c^	1.13	55.45 ± 21.74
	Placebo	36.80 ± 17.34	30.33 ± 14.66	28.80 ± 12.61	< 0.001^a^				21.74 ± 14.74
ASI	Intervention	22.04 ± 9.96	11.11 ± 6.09	7.42 ± 5.44	< 0.001^a^	0.038^b^	0.203^c^	1.02	66.33 ± 27.15
	Placebo	19.52 ± 9.83	15.41 ± 8.66	14.22 ± 7.71	< 0.001^a^				27.15 ± 15.42

^a^ *p*‐values derived from repeated measures ANOVA for within‐group comparisons over time.

^b^ *p*‐values derived from repeated measures ANOVA for between‐group comparisons over time.

^c^ *p*‐values derived from repeated measures ANOVA for between‐group comparisons over time after adjusted by age and education.

^d^ Percent change between time of beginning and 8th week.

**FIGURE 2:**
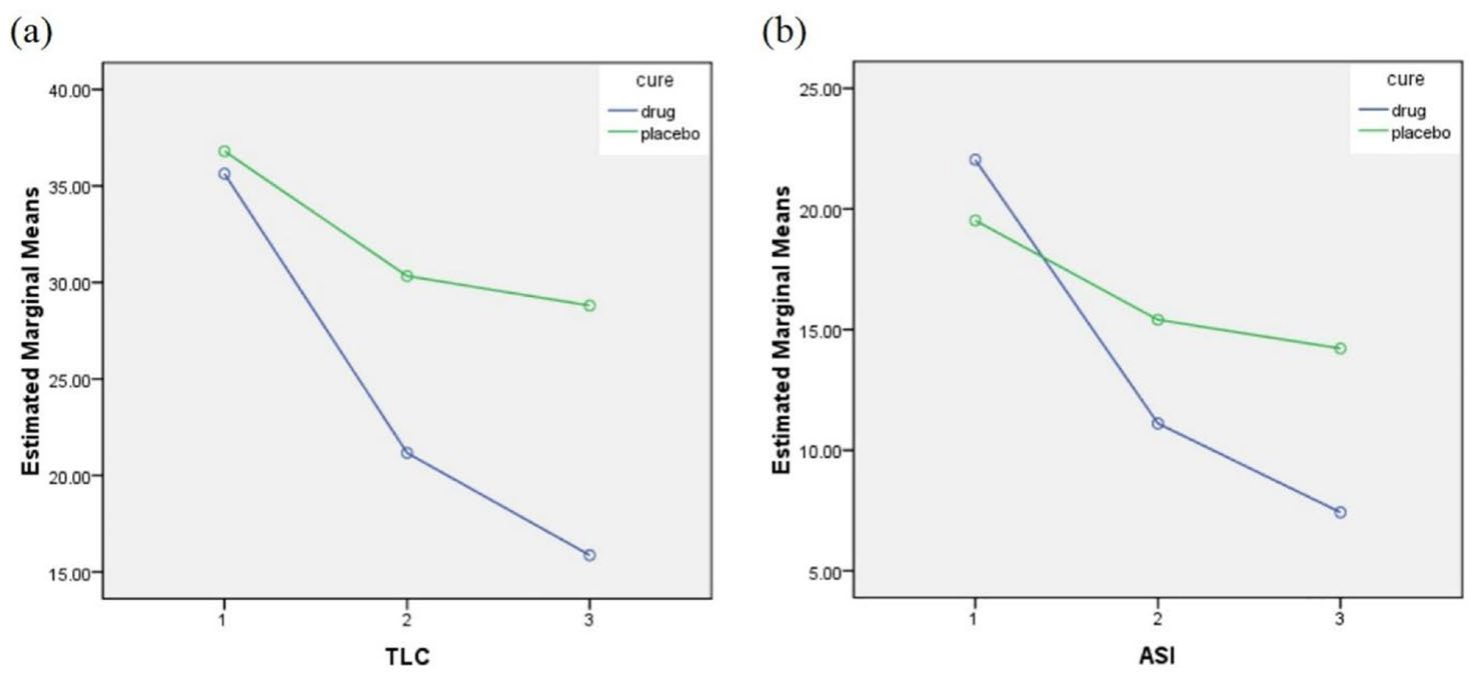
(a) Mean TLC over time in intervention and placebo groups, (b) changes in ASI over time between the intervention and placebo groups.

Figure 2a illustrates the trend in TLC reduction over the study period. In the intervention group, a steep decline in TLC was observed from baseline to the 8th week, with a significant reduction evident by the 4th week. The placebo group showed a slower and less pronounced reduction in TLC, with a plateauing effect after the 4th week. These trends emphasize the enhanced effectiveness of the intervention compared to the placebo (Figure 2a).

Figure 2b depicts the changes in ASI across the study timeline. The intervention group showed a consistent and significant reduction in ASI. The most marked improvement occurs between the baseline and the 4th week, followed by a continued but slower decline up to the 8th week. Conversely, the placebo group exhibited a gradual and less significant reduction, with minimal changes between the 4th and 8th weeks. This pattern highlights the superior therapeutic effect of the intervention on acne severity (Figure 2b).

### 3.3 Secondary Outcomes

Table 3 presents the distribution of patient satisfaction levels between the two groups. In the intervention group, 43.3% of patients reported being very satisfied with the intervention, compared to only 3.3% in the placebo group (*p* < 0.001). Dissatisfaction rates were significantly higher in the placebo group, with 20% reporting being very dissatisfied, compared to 3.3% in the intervention group (*p* < 0.001). This data indicates a significantly higher level of satisfaction among patients using the intervention which supports its potential for better patient adherence and satisfaction.

**TABLE 3:** Comparison of patient satisfaction with drug usage between intervention and placebo groups.

Level of satisfaction	Intervention [*n* (%)]	Placebo [*n* (%)]	*p*
Very dissatisfied	1 (3.3)	6 (20.0)	0.001^a^
Somewhat dissatisfied	0 (0.0)	10 (33.3)	
Neutral	4 (13.3)	4 (13.3)	
Somewhat satisfied	12 (40.0)	9 (30.0)	
Very satisfied	13 (43.3)	1 (3.3)	

^a^ *p*‐values calculated using Fisher's Exact test.

### 3.4 Adverse Events

Adverse events were mild and predominantly reported in the placebo group, including transient skin irritation in 10% of participants. In the intervention group, adverse events were minimal, with no reported cases of severe reactions. These findings highlight the tolerability of the intervention.

## 4 Discussion

The findings of this study highlight the remarkable potential of a natural facial mask combining Bentonite and hydroalcoholic extract of *Alcea sulphurea* in managing moderate acne. Compared to the placebo group, participants using this formulation experienced significantly greater reductions in total lesion count and acne severity index over an eight‐week period. Furthermore, patient satisfaction was notably higher in the intervention group. This reflects both the visible improvements in skin condition and the tolerability of the treatment. These results suggest that integrating traditional medicine principles with modern clinical practices can yield innovative, effective, and patient‐friendly approaches to acne management, which provide a promising alternative to conventional treatments. Acne vulgaris remains one of the most common dermatological conditions. It significantly impacts patients' quality of life, self‐esteem, and psychological well‐being. Its complex etiology, involving sebaceous hyperactivity, microbial colonization (particularly by *Cutibacterium acnes*), inflammation, and hormonal imbalances, requires a multifaceted therapeutic approach [20, 21].

The intervention group demonstrated a significant reduction in TLC and ASI over the study period, outperforming the placebo group. Specifically, the mean percent reduction in ASI in the intervention group was 66.33%, compared to 27.15% in the placebo group. These results underscore the therapeutic potential of the facial mask.

Bentonite, a natural clay, is rich in minerals and has been traditionally used for its absorbent, exfoliative, and detoxifying properties. By absorbing excess sebum, unclogging pores, and removing impurities, Bentonite directly addresses some of the root causes of acne, such as sebaceous hyperactivity and comedone formation [22, 23]. *A. sulphurea* extract, complements these effects with its potent anti‐inflammatory and hydrating properties. The mucilage content in *Alcea sulphurea* extract soothes irritated skin and reduces inflammation, which is a critical component of acne pathogenesis [24, 25]. Previous studies have highlighted the benefits of clay‐based masks in treating acne. The results were similar to those observed in this study [26, 27, 28]. For example, research on mineral clays has shown improvements in skin texture, reduced oiliness, and minimized inflammation [29, 30, 31]. While *Alcea sulphurea* extract has not been extensively studied in acne management, its emollient properties are well documented in treating skin irritation and inflammation [32, 33]. Interestingly, the synergistic combination of Bentonite and *Alcea sulphurea* extract in this study appears to have amplified their individual benefits, as evidenced by the substantial improvements in TLC and ASI. While previous research has established the benefits of clay‐based masks, this study extends the scope by integrating an herbal extract with anti‐inflammatory properties. This paves the way for more innovative and patient‐friendly formulations. Future studies could focus on elucidating the exact mechanisms behind the synergy observed in this combination and exploring its efficacy in broader and more diverse populations.

Patient satisfaction is a critical yet often overlooked outcome in acne studies. In this study, 43.3% of patients in the intervention group reported being very satisfied, compared to only 3.3% in the placebo group. Higher satisfaction rates in the intervention group likely stem from the visible improvements in skin condition, minimal side effects, and the pleasant application experience of the mask. Satisfaction not only reflects the perceived effectiveness of a treatment but also influences adherence. It is crucial for achieving sustained clinical benefits. The high satisfaction rates observed in this study suggest that the Bentonite and *A. sulphurea* extract mask could improve adherence, which leads to sustained clinical benefits over time.

This study has several strengths. Its randomized design ensured balanced allocation of participants, minimizing selection bias. Additionally, the comprehensive evaluation of both objective clinical outcomes and subjective patient‐reported outcomes provides a holistic understanding of the mask's effectiveness. The use of natural ingredients, inspired by traditional medicine, offers a promising alternative to conventional treatments, especially for patients seeking holistic and tolerable therapies [34].

By bridging traditional and modern approaches, this study lays the groundwork for integrating natural, patient‐friendly treatments into acne management protocols, which potentially reduce reliance on conventional therapies with known side effects.

This study faced several limitations that should be acknowledged. First, the sample size was relatively small, consisting of only 60 participants, which may limit the generalizability of the findings to a larger population. One of the possible limitations of this study was that, given the fact that there was no similar study at the time of the proposal to estimate the sample size, 30 samples were included in each group using the Central Limit Theorem. Recruitment challenges also extended the timeline for patient enrollment, as identifying individuals meeting the inclusion criteria proved time‐consuming. Additionally, the short duration of the study (8 weeks) precluded the evaluation of the long‐term efficacy and safety of the intervention. Future studies with larger sample sizes and extended follow‐up periods are necessary to confirm these results and explore potential long‐term benefits and risks. Another limitation was the inability to completely blind participants due to the physical differences between the intervention (mask) and placebo (gel) formulations. This could have introduced bias in patient‐reported outcomes, such as satisfaction levels. Although we used randomization, allocation concealment, opaque packaging and standardized instructions, the difference in the product format may nonetheless have allowed some participants to deduce their group assignment, potentially affecting their subjective perceptions and responses. We suggest that future studies consider matched placebo formats or other strategies to strengthen blinding and reduce the residual risk of bias.

Future research should aim to include participants with a wider range of skin types and backgrounds to enhance the robustness of the conclusions. Additionally, exploring the therapeutic effects of the mask in other inflammatory skin conditions, such as rosacea or eczema, could provide further evidence of its versatility. Despite these limitations, the study provides valuable insights into the potential of traditional medicine‐based formulations for acne management, laying the groundwork for further research in this area.

## 5 Conclusion

The study provides evidence supporting the effectiveness of a facial mask based on Bentonite and hydroalcoholic extract of *A. sulphurea* in managing moderate acne. The intervention significantly reduced lesion counts and acne severity while achieving higher patient satisfaction compared to the standard treatment with Azithromycin and placebo. These findings highlight the potential of natural, traditional medicine‐based formulations as effective and patient‐friendly alternatives for acne management. Further research is warranted to confirm these results and explore additional therapeutic applications.

## Author Contributions

Sara Salimi: writing the original draft, methodology and investigation, principal investigator. Moloud Fakhri: methodology and investigation. Majid Saeedi and Emran Habibi: supervision, formulation scientist. Seyyed Mobin Rahimnia: supervision, formulation scientist, reviewing, and editing of the manuscript. Armaghan Kazeminejad: supervision, data analysis. Mahmood Moosazadeh: statistical analysis. Assie Jokar: supervision, conceptualization and study design, reviewing, and editing of the manuscript.

## Funding

This work was supported by Mazandaran University of Medical Sciences.

## Ethics Statement

The authors confirm that all experiments were performed in accordance with relevant guidelines and regulations. Also, they confirm that informed consent was obtained from all subjects. All the methods/study were in accordance with the declaration of Helsinki, and all methods were conducted in accordance with relevant guidelines and regulations. This add‐on randomized controlled clinical trial was approved by the Committee of Medical Ethics, Mazandaran University of Medical Science (approval code: IR.MAZUMS.REC.1400.683), and was registered by the Iranian Registry of Clinical Trials (registration ID: IRCT20220403054395N1).

## Conflicts of Interest

The authors declare no conflicts of interest.

## Supporting information


**Appendix S1:** jocd70586‐sup‐0001‐Supinfo.docx.

## Data Availability

The data that support the findings of this study are available on request from the corresponding author. The data are not publicly available due to privacy or ethical restrictions.
