# Supplementary material for: The Therapeutic Effects of a Bentonite‐Based Facial Mask With Alcea sulphurea Extract on Acne Severity and Patient Experience: Add‐On Randomized Controlled Clinical Trial
Source: J Cosmet Dermatol. 2025 Dec 5;24(12):e70586. doi: 10.1111/jocd.70586 (PMC12679517; doi:10.1111/jocd.70586)

The efficacy of Bentonite-based facial mask with *Alcea sulphurea* extract on acne severity. Below are some patient’s photos before and after the intervention.

1. Before


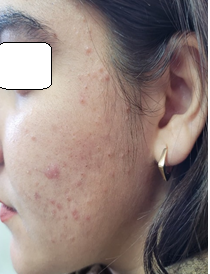

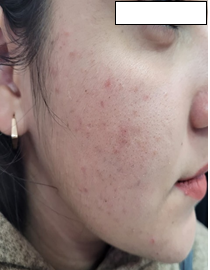


1. After


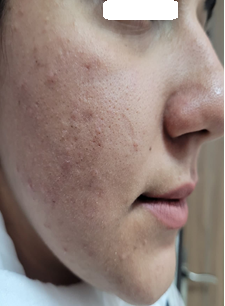

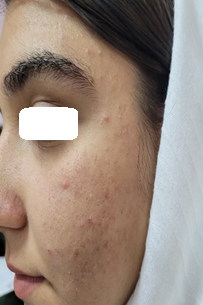


2. Before


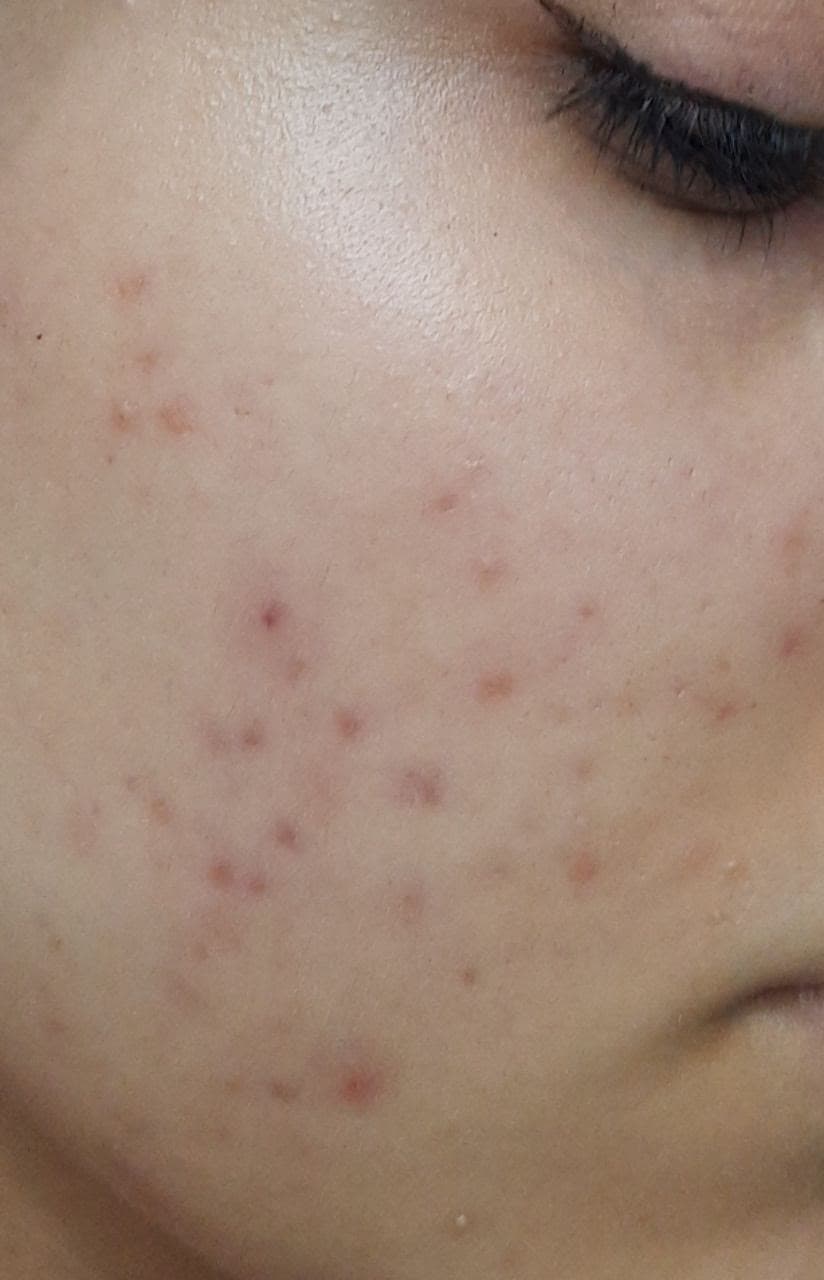

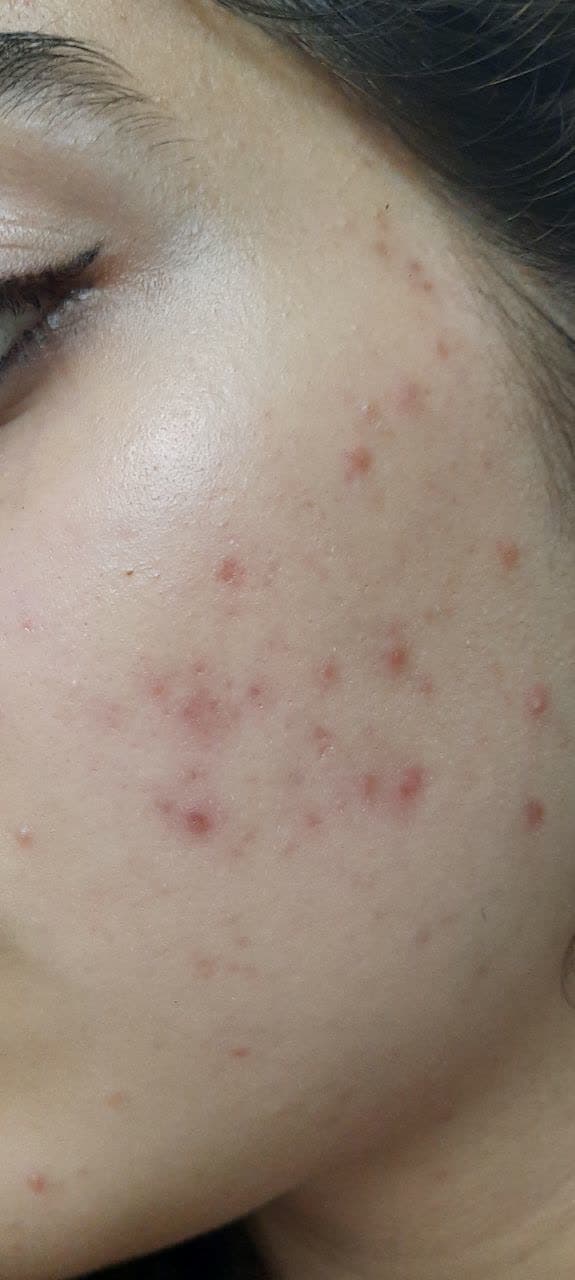

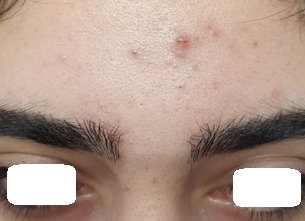


1. After:


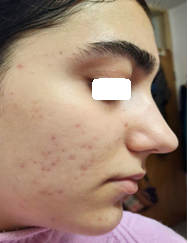

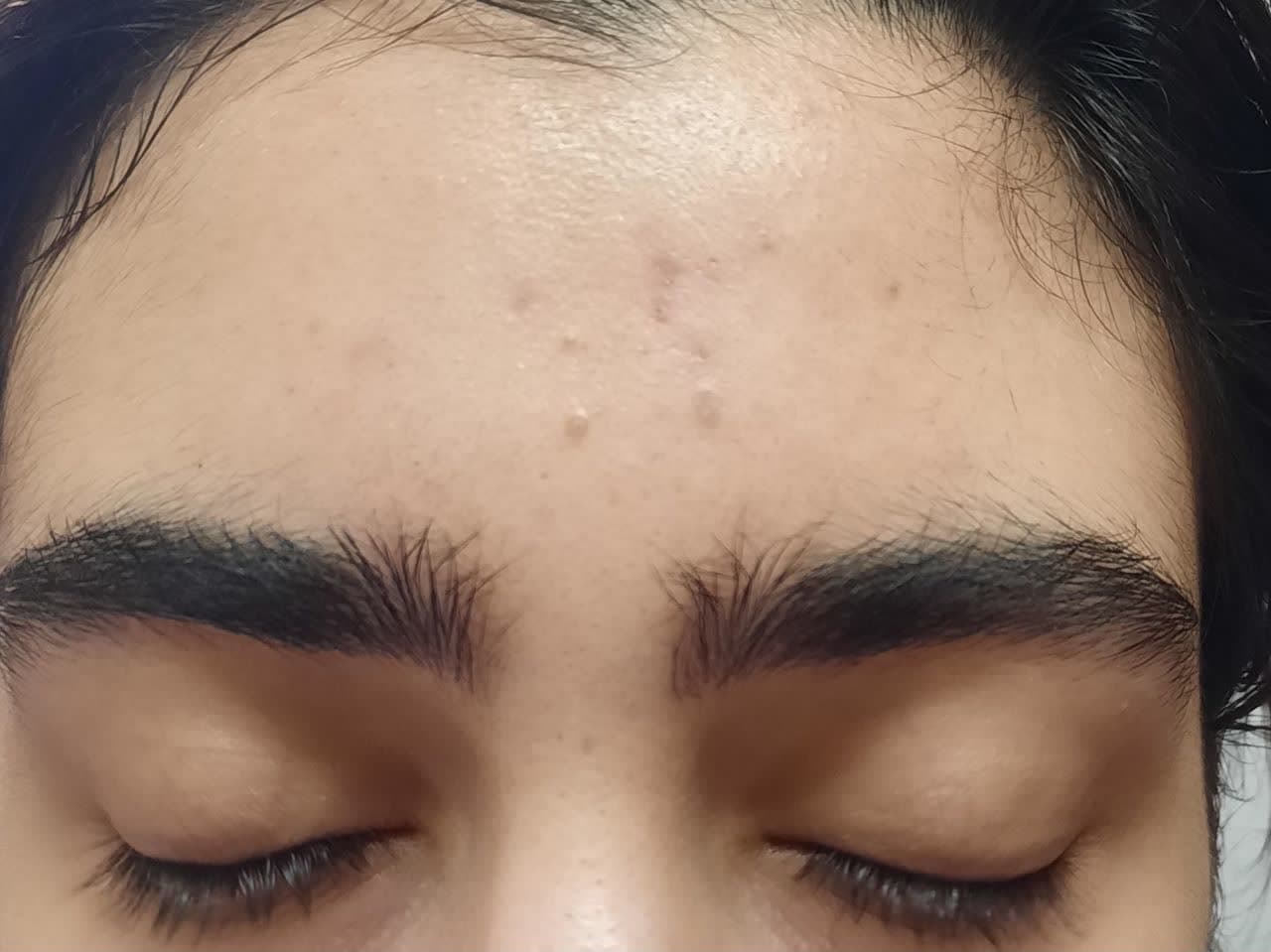

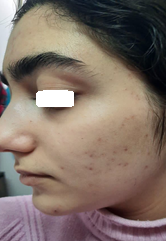


3. Before:


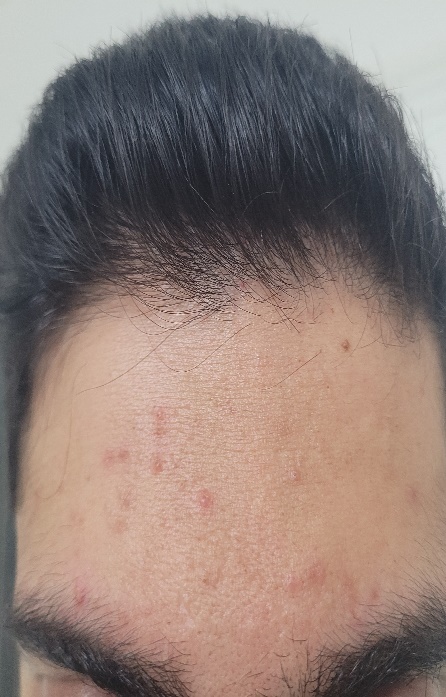

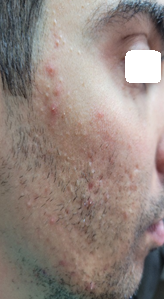

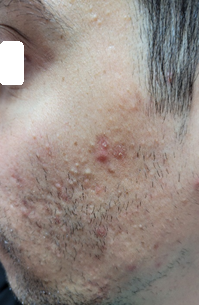


3. After:


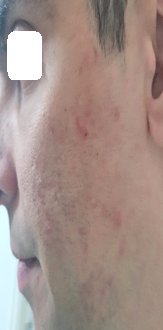

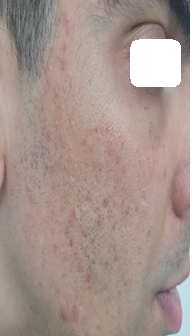

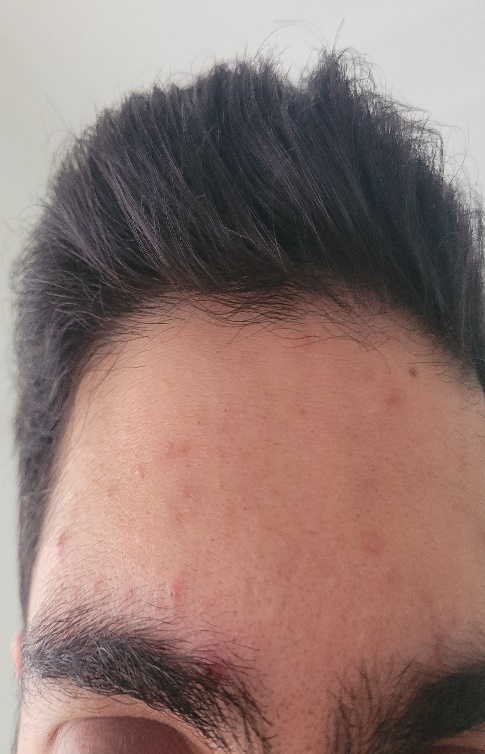


4. Before


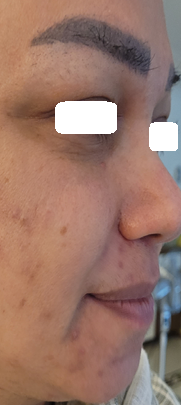

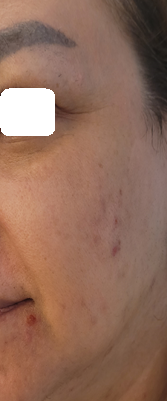

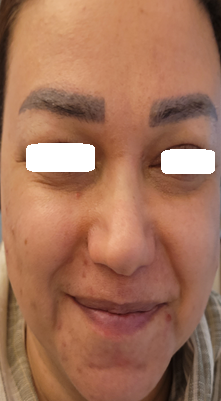


4. After


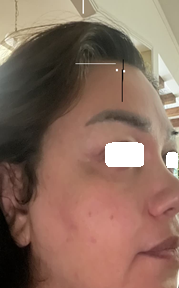

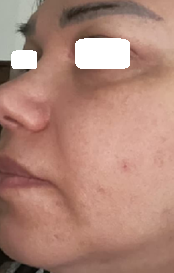

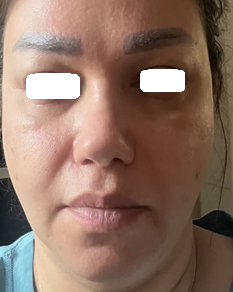

Supplement: Supplementary file 1 — Appendix S1: jocd70586‐sup‐0001‐Supinfo.docx. [file JOCD-24-e70586-s001.docx]
